# Contribution of Aberrant Toll Like Receptor Signaling to the Pathogenesis of Myelodysplastic Syndromes

**DOI:** 10.3389/fimmu.2020.01236

**Published:** 2020-06-17

**Authors:** Luana Chiquetto Paracatu, Laura G. Schuettpelz

**Affiliations:** Department of Pediatrics, Washington University School of Medicine, St. Louis, MO, United States

**Keywords:** TLR—toll-like receptor, pyroptosis, cell death, inflammation, myelodysplastic syndromes

## Abstract

Toll like receptors (TLRs) are a family of pattern recognition receptors that play a central role in the innate immune response. These receptors are expressed on a wide variety of immune and non-immune cells, and they help shape the immune response to infection and injury through the recognition of pathogen-associated molecular patterns (PAMPs) as well as endogenous damage-associated molecular patterns (DAMPs). Accumulating evidence suggests that, in addition to regulating mature effector immune cells, TLRs can influence the immune response from the level of the hematopoietic stem cell (HSC). HSCs express TLRs, and exposure to TLR ligands influences the cycling, differentiation, and function of HSCs, with chronic TLR stimulation leading to impairment of normal HSC repopulating activity. Moreover, enhanced TLR expression and signaling is associated with myelodysplastic syndromes (MDS), a heterogenous group of HSC disorders characterized by ineffective hematopoiesis and a high risk of transformation to acute leukemias. In this review, we will discuss the role of TLR signaling in the pathogenesis of MDS, focusing on the known direct and indirect effects of this type of signaling on HSCs, the mechanisms of TLR signaling upregulation in MDS, the changes in TLR expression with disease progression, and the therapeutic implications for modulating TLR signaling in the treatment of MDS.

## Introduction

The TLRs are a family of pattern recognition receptors that recognize foreign pathogen-associated molecular patterns (PAMPs) as well as endogenous byproducts of cellular damage (so called “damage-associated molecular patterns,” or DAMPs) (1, 2), and play a central role in the innate immune response to infections and tissue injury (3–6). TLR signaling regulates not only mature effector cells of the immune system, but also influences hematopoietic stem cells (HSCs) through both cell-autonomous and cell non-autonomous mechanisms (7–9). Furthermore, chronic TLR signaling can impair normal HSC function (10). In patients with myelodysplastic syndromes (MDS), TLR signaling is enhanced via a combination of genetic and epigenetic events that influence multiple components of the TLR signaling pathway. This enhanced signaling is particularly notable in lower-risk MDS, and contributes to the marked cell death and ineffective hematopoiesis characteristic of this disease (11). Here, we focus on the role of TLR signaling in MDS, highlighting the mechanisms and implications of TLR upregulation, the differences between lower- and higher-risk disease, and the therapeutic opportunities for targeting TLR signaling in the treatment of MDS.

### TLR Signaling Regulates Hematopoietic Stem and Progenitor Cell Function

The TLR family is comprised of 10 members in humans (TLR1-TLR10) (5, 12–15), and 12 members in mouse (TLR1- TLR9, and TLR11-TLR13) (16). TLRs are expressed on a variety of hematopoietic cell types, such as dendritic cells, macrophages, and lymphocytes, as well as non-hematopoietic cells such as endothelium and epithelium (17–20), and can be found at the cell surface (TLR1, TLR2, TLR4, TLR5, TLR6, and TLR11) or within endosomes (TLR3, TLR7, TLR8, and TLR9). TLRs largely function as homodimers, with the exception of TLR2, which heterodimerizes with either TLR1 or TLR6 (21–23). With the exception of TLR3, which signals via the adaptor TIR-domain-containing adaptor-inducing interferon-β (TRIF) (24, 25), the TLRs utilize the intracellular signaling adaptor myeloid differentiation primary response gene 88 (MyD88) (26). MyD88 is recruited to activated TLRs, and this is followed by recruitment of members of the serine-threonine kinase interleukin-1 receptor-associated kinase (IRAK) family (IRAK4, IRAK1, and IRAK2), forming the so-called “Myddosome.” (27–29) Activated IRAK1 then promotes the activity of the E3 ubiquitin ligase tumor necrosis factor (TNF) R-associated factor 6 (TRAF6), which in turn activates transforming growth factor beta-activated kinase 1 (TAK1) leading to stimulation of mitogen activated protein kinase (MAPK) and nuclear factor k-light-chain-enhancer of activated B cells (NF-kB) pathways and the production of proinflammatory cytokines (30). Endosomal TLRs (TLR3, TLR7, TLR8, and TLR9) act via the Myddosome and TRAF6 to activate IFR7 and promote type I interferon production. TRIF-dependent TLR signaling, utilized by TLR3 and, in part, TLR4, involves the binding of TRIF to TRAF3, which then recruits TBK1 and IKKε thereby stimulating IRF3 and also promoting the production of type I interferons (31–34).

In addition to regulating mature immune effector cells, TLRs are expressed on more primitive hematopoietic stem and progenitor cells (HSPCs) (7, 20, 35, 36), and even in the absence of overt infection or injury, signaling through the TLRs influences the differentiation, and function of HSPCs. Proinflammatory signals, including TLR4-MyD88-NF-kB activation, are required for HSC specification in both zebrafish and mouse embryos (37, 38). Thereafter, TLR signaling continues to regulate HSCs, as the bone marrow from mice deficient in TLR signaling (*Tlr2*^−/−^, *Tlr4*^−/−^, *Tlr9*^−/−^, and *MyD88*^−/−^ mice) display enhanced repopulating activity in competitive transplantation experiments (39, 40). Conversely, chronic exposure to TLR ligands is associated with a loss of normal HSC function. Persistent exposure of mice to the TLR4 ligand LPS, for example, is associated with an expansion of immunophenotypic HSCs, but a loss of HSC repopulating function in transplantation experiments (10). Similarly, we recently demonstrated that treatment of mice with the TLR1/2 ligand PAM_3_CSK_4_ expands immunophenotypic HSCs in both the bone marrow and spleen, but reduces their repopulating activity (8). Studies in chimeric mouse and *in vitro* models suggest that these effects of TLR ligands on HSCs are mediated by both direct (cell autonomous) and indirect (cell non-autonomous) mechanisms. Supporting a direct effect of TLR signaling on HSCs, for example, TLR stimulation via LPS or PAM_3_CSK_4_ leads to proliferation and myeloid differentiation of sorted HSPCs (Lineage- c-Kit+ Sca-1+ cells) in culture (7), and purified HSPCs from wild-type mice transplanted into TLR-deficient (*Tlr2*^−/−^, *Tlr4*^−/−^, or *MyD88*^−/−^) recipients (thus minimizing indirect effects of TLR stimulation) differentiate into macrophages upon treatment with TLR ligands (9). Takizawa et al. (41) used chimeric mice (*Tlr4*^−/−^ and WT cells transplanted into *Tlr4*^−/−^ recipients) to show that LPS directly stimulates HSC proliferation and impairs HSC self-renewal. Further, they found that the LPS-induced loss of HSC self-renewal is TRIF-, and not MyD88-, dependent, and is mediated by the production of reactive oxygen species (ROS) and activation of p38 MAPK (41). Zhang et al. (42) similarly showed that LPS-induced loss HSC self-renewal is TRIF-dependent, and further highlighted the differential effects of TRIF- and MYD88- dependent TLR4 signaling on HSPCs. While TRIF-mediated signaling accounts for HSC expansion and loss of self-renewal in response to LPS, MYD88- dependent signaling largely accounts for LPS-induced myelosuppression via impaired differentiation and increased death of myeloid progenitors (42). Our own studies using chimeric mice treated with the TLR1/2 agonist PAM_3_CSK_4_ showed that while the loss of HSC function in response to this type of stimulation is largely cell non-autonomous, direct signaling does contribute to the expansion of immunophenotypic HSCs (8). Thus, multiple TLRs can influence HSCs, both via direct and indirect mechanisms.

### TLR Expression and Signaling Is Increased in Patients With Myelodysplastic Syndromes (MDS), and Is Associated With Disease Severity

The myelodysplastic syndromes (MDS) are a group of hematopoietic stem cell disorders characterized by abnormal hematopoiesis and a high risk of transformation to acute myeloid leukemia (AML) (43, 44). There are ~10,000 new cases of MDS per year in the US, and the incidence increases with age (45). MDS is rare in children and young adults, though it may occur in these populations in association with inherited bone marrow failure syndromes (46, 47). Patients with MDS suffer from cytopenias, requiring supportive care (e.g., red blood cell and platelet transfusions), and risk life-threatening consequences of these cytopenias such as infections and bleeding. In addition, about 30% of patients with MDS will progress to develop AML (48). The prognosis of patients with MDS is determined using the revised International Prognostic Scoring System (IPSS-R), which considers the degree of cytopenias, bone marrow blast percentage, and cytogenetic abnormalities (49). This score is used to place patients into lower- and higher-risk categories, and helps to inform their treatment. Depending on the age of the patient, risk category, and other comorbid factors, the treatment options include supportive care, hypomethylating agents, immunomodulatory drugs, and allogeneic hematopoietic stem cell transplantation (50). Stem cell transplantation is the only curative option, however this is often not feasible due to patient age or other comorbid factors (51). Thus, new therapies are needed. As discussed below, targeting of innate immune signaling, and particularly TLRs, is an attractive new therapeutic approach.

The pathogenesis of MDS is thought to involve a combination of genetic mutations (particularly involving genes that regulate RNA splicing or influence the epigenetic landscape, in addition to variety of other oncogenes, and tumor suppressors) (52–55), which may be induced or selected for by genotoxic insults, inflammatory conditions, and/or aging, and hyper-activation of innate immune signaling pathways as a result of these genetic and epigenetic changes that contribute to HSPC senescence, impaired differentiation and cellular death (56–59). In lower-risk MDS, intact DNA damage checkpoints and pro-death signals lead to the prominent intramedullary cell death characteristic of lower-risk disease (60, 61). In contrast, the presence of mutations that confer resistance to these damage checkpoints and pro-death signals is characteristic of higher-risk MDS with an accelerating clonal expansion and increased risk of transformation to acute leukemia (62, 63). Notably, recent studies on the clonal evolution of MDS suggest that in many cases, disease progression is non-linear, with diverse subclones evolving in a parallel fashion to give rise individually to the development of MDS and secondary AML (62, 64, 65).

Regarding the contribution of hyper-activated innate immune signaling, numerous studies have shown that TLR signaling is enhanced in the CD34+ HSPCs of patients with MDS, with multiple components of the pathway (TLRs themselves or downstream effectors) either overexpressed or aberrantly activated compared to healthy controls (66–70). Wei et al. (69) profiled the mRNA expression of eight TLRs (TLR1-4 and TLR6-9) in a cohort of 149 patients with MDS, and found increased expression of TLR2 and its binding partners, TLR1 and TLR6, in the CD34+ cells of these patients compared to healthy controls. Notably, they showed that TLR2 expression was highest in the lower-risk risk patients, correlating with better overall survival, and conversely, TLR6 expression was highest in the higher-risk patients. Zeng et al. (70) similarly found that TLR2 was more highly expressed in the CD34+ cells of patients with lower-risk MDS compared to healthy controls or to patients with high-risk disease, and they noted that TLR2 expression correlated with increased apoptosis. Further, they showed that TLR2 agonist-associated CD34+ cell death was mediated by β-arrestin1 upregulation and histone H4 acetylation via recruitment of P300. β-arrestin1 mRNA levels and nuclear accumulation of β-arrestin1 protein were significantly increased in the CD34+ cells of patients with low-risk disease compared to higher-risk MDS or healthy controls, and inhibition of β-arrestin1 in cultured CD34+ cells mitigated TLR2 agonist-induced cell death (70). In a smaller series of 21 patients, Maratheftis et al. (68) reported increased expression of TLR4 in the CD34+ cells of patients with MDS, and this expression also correlated with cell death. In addition to these TLRs, mRNA expression of the downstream mediator of TLR signaling, MyD88, was found to be increased in the CD34+ cells of patients with MDS compared to healthy controls, and inhibition of MyD88 in cultured CD34+ cells of lower-risk patients led to increased erythroid colony formation. Of note, like the TLRs, this expression associated with disease status, with lower-risk patients displaying higher MyD88 expression than higher-risk patients (67).

Not only are the TLRs overexpressed in MDS, but multiple genetic abnormalities and epigenetic events in MDS have been found to contribute to enhanced sensitivity to TLR ligands. In patients with deletions of 5q (del(5q) MDS), for example, loss of a copy of the micro-RNAs mir-145 and miR-146a result in activated TLR signaling. These micro-RNAs normally inhibit the TLR signaling intermediates TIRAP (mir-145) and TRAF6 (mir-146a), and knockdown of these miRs or enforced expression of TRAF6 in mouse hematopoietic stem and progenitor cells recapitulates some of the features of human del(5q) MDS including elevated serum IL-6 levels, megakaryocytic dysplasia, and progression to marrow failure or acute myeloid leukemia (71). Enhanced TRAF6 activity in del(5q) MDS is further exacerbated by haploinsufficiency for another 5q gene, TRAF-interacting protein with forkhead-associated domain B (TIFAB), which normally promotes TRAF6 degradation via a lysosome-dependent mechanism (72). TRAF6, a ubiquitin ligase, contributes to impaired hematopoiesis and bone marrow failure via ubiquitination of the RNA-binding protein and auxiliary splicing factor hnRNPA1. This modification of hnRNPA1 results in alternative splicing and reduced expression of Arhgap1, an inhibitor of the GTP-binding Rho family protein Cdc42, which in turn leads to aberrant hematopoiesis (73). Very recently, Muto et al. (74) showed that TRAF6- overexpressing HSPCs have a competitive advantage over their normal counterparts in the face of low-dose LPS (TLR4 ligand). This advantage is dependent upon the expression of A20 and the activation of non-canonical NF-κB signaling, suggesting that the mechanistic basis for the clonal advantage of MDS HSPCs involves an adaptive response to TLR stimulation with a shift from canonical to non-canonical NF-κB signaling.

Additionally, spliceosome mutations (e.g., involving spliceosome components such as SF3B1, U2AF1, and SRSF2), which are common in MDS (75), have been found to confer sensitivity to TLR ligands by promoting the production of activating forms of components of the TLR signaling pathways. For example, Smith et al. (76) found that the expression of a longer isoform of IRAK4 (IRAK4-L), which is associated with enhanced activation of NF-kB and leukemic transformation, is mediated by mutant forms of the splicing factor U2 small nuclear RNA auxiliary factor 1 (U2AF1). Similarly, Alper et al. (77–79) showed that inhibition of spliceosome genes reduces inflammatory cytokine production in response to TLR agonists, and conversely, expression of MDS-associated spliceosome mutations (involving splicing factors U2AF1, SF3B1, or SRSF2) enhances NF-κB activity and cytokine production by K562 myeloid leukemia cells following TLR stimulation. Mechanistically, the spliceosome mutations alter the splicing of genes that regulate TLR signaling and promote NF-κB activation, including MAP3K7 and CASP8 (77).

In addition to increased expression of TLRs and components of the TLR signaling pathways, as well as enhanced sensitivity to TLR ligands in the HSPCs of patients with MDS, some of the known TLR ligands are more abundant in the bone marrow and/or serum of individuals with MDS compared to healthy controls. High mobility group box 1 (HMGB1), for example, a nuclear DAMP released upon cell death that stimulates TLR2 and TLR4, is increased in the serum of patients with MDS (80). Kam et al. (81) recently reported that HMGB1 inhibition in cultures of an MDS cell line or primary MDS CD34+ cells with the small molecule sivelestat reduced cellular expansion and colony forming ability *in vitro*, as well as engraftment of the MDS-L cell line into NSG mice. Further, inhibition of HMGB1 resulted in enhanced cell death, with increased expression of p53-upregulated modulator of apoptosis (PUMA) and activation of caspase-3. Interestingly, this inhibition also reduced the expression of TLRs themselves in MDS CD34+ cells, suggesting that TLR stimulation may amplify its own expression in MDS.

Besides HMGB1, the TLR4 ligands S100A8 and S100A9 are elevated in the bone marrow and blood of MDS patients compared to controls, with the highest levels specifically found in patients with lower-risk disease (82, 83). S100A8 and S100A9 are members of the S100 family of calcium-binding proteins that exist as heterodimers (known as calprotectin) or homodimers, and are expressed constitutively by myeloid lineage cells (84, 85). These endogenous DAMPS are passively released by necrotic cells or actively secreted by activated immune cells (86), bind to both TLR4 and the myeloid cell receptor CD33, and contribute to the ineffective hematopoiesis in MDS via direct and indirect effects on HSPCs (87). S100A8 and S100A9, in addition to playing a direct role in promoting cell death in HSPCs in MDS, as discussed further below, expand myeloid derived suppressor cells (MDSCs) in the bone marrow of patients with MDS through binding to CD33. MDSCs are CD33+ Lineage- HLA-DR- immature myeloid cells, and their frequency is significantly increased in patients with MDS compared to healthy controls (88, 89). Supporting a role for MDSCs and S100A9 in the pathogenesis of MDS, S100A9 transgenic mice that overexpress this DAMP in hematopoietic cells display an accumulation of MDSCs with age, and develop progressive multilineage cytopenias and dysplasias characteristic of human MDS (88). MDSCs, which are typically distinct from the malignant clone in MDS, contribute to the suppression of normal hematopoiesis via the production of cytokines such as IL-10 and transforming growth factor beta (TGFβ) (89). In addition, MDSCs can exert a direct suppressive effect on the growth of erythroid and myeloid progenitor cells *in vitro* (88), and very recent data from Cheng et al. showed that the immune checkpoint receptor programmed cell death protein-1 (PD-1) and its ligand programmed cell death-ligand 1 (PD-L1) are upregulated in response to S100A9 signaling and contribute to MDSC-induced HSPC death in MDS (90). Activated MDSCs secrete more S100A8 and S100A9 (91), thus supporting autocrine and paracrine TLR and CD33 stimulation and promoting further expansion of MSDCs and inflammatory cytokine production.

Together, the above studies clearly establish enhanced TLR signaling as a common feature of MDS, particularly in the context of lower-risk disease. The role of this enhanced TLR signaling in the pathogenesis of the disease remains somewhat unclear, however, and additional studies are necessary to fully elucidate the contribution of TLR signaling to the initiation and promotion of MDS. In addition, the etiology of increased TLR expression in MDS is not well-understood, and further studies are warranted to determine how TLR expression is regulated in both lower- and higher-risk MDS HSPCs. TLR expression and signaling patterns differ between low- and high-risk MDS, and the origin of these differences and the roles of specific TLRs in different stages of disease are not clear. Notably, TLR expression is induced in a variety of cell types by inflammatory cytokines (92–94) and also via positive feedback by TLR ligation itself (81), suggesting that the inflammatory microenvironment may reinforce elevated TLR expression and signaling. Finally, studies of TLR signaling in MDS have largely focused on HSPCs, and, aside from MDSCs, the expression of TLRs and contribution of TLR signaling in populations outside of HSPCs to the pathogenesis of MDS is not well-understood. One aspect of MDS pathogenesis that is clearly influenced by TLR signaling is cell death, with accumulating data, as discussed below, implicating this signaling in promoting the intramedullary death of HSPCs in patients with lower-risk MDS.

### TLR Signaling Promotes Cell Death in MDS

A hallmark of low-risk MDS is increased death of bone marrow CD34+ cells (95–98), and multiple studies have implicated a role for TLR signaling in this process. Both TLR2 and TLR4 signaling have been associated with cell death in MDS, and recent studies demonstrate that TLR signaling promotes an inflammatory form of programmed cell death termed pyroptosis (11, 99, 100). Pyroptosis is mediated by the formation of multiprotein complexes, known as the inflammasome, composed of nucleotide-binding domain and leucine-rich repeat pattern-recognition receptors (NLRs), most notably NOD-like receptor pyrin domain-containing 3 (NLRP3) (101). NLRP3 assembly, which is stimulated by DAMPs, leads to the recruitment of the adaptor protein apoptosis-associated speck like protein containing a CARD (PYCARD, also known as ASC), which then form large helical fibrils that assemble into complexes referred to as ASC specks (102–104). These specks function as a platform for the binding and autoproteolytic activation of pro-caspase-1, which then activates pro-IL-1β and pro-IL-18 as well as the pore-forming protein gasdermin D (105–111). Membrane pores created by activated gasdermin D lead to cation entry and cell swelling, triggering pyroptotic cell death with the release of active IL-1β, IL-18, and as well as ASC specks and other intracellular proteins that contribute to local inflammation (112, 113).

TLR agonists such as S100A8 and S100A9, which bind to TLR4 and CD33, stimulate inflammasome assembly, and pyroptosis by enhancing the production of inflammasome components, and proinflammatory cytokines such as pro-IL-1β, and pro-IL-18. In addition, TLR agonists promote the activation of NADPH oxidase to generate reactive oxygen species (ROS), which contribute to inflammasome assembly and inflammatory cytokine production (114). In a recent study, Basiorka et al. (82) reported that markers of pyroptosis (activated caspase-1, NLRP3, and ASC speck levels) were higher in the bone marrow mononuclear cells (BM-MNCs) of patients with MDS compared to healthy controls, and cell death was inhibited by shRNA-mediated knockdown of NLRP3 or caspase- 1. Further, they showed that S100A9- induced signaling activates NADPH oxidase, increasing ROS and triggering pyroptosis and β-catenin activation in MDS BM-MNCs, and inhibition of S100A9 using a decoy receptor reduced transcriptional priming of pyroptosis-associated genes (CASP1, IL-1B, IL-18, and NLRP3) and improved the colony-forming capacity of BM-MNCs from patients with MDS. In a follow up survey of 177 patients with MDS (115), Basiorka et al. quantified ASC specks by flow cytometry and found the mean percentage of peripheral blood plasma-derived ASC specks to be significantly higher than in 29 age-matched healthy controls (115). These findings were validated in an additional cohort of 133 patients and 31 healthy controls, and they found that ASC speck percentage correlated with lower-risk disease as well as with S100A8 and S100A9 plasma levels. These data support the idea that pyroptosis is a prominent form of cell death in MDS, with TLR signaling- particularly stimulation via S100A8 and S100A9- contributing to this process.

Further implicating TLR signaling in the promotion of pyroptotic cell death in MDS, we recently reported that loss of TLR2 or MyD88 in the *NUP98-HOXD13* (*NHD13*) mouse model of MDS led to a reduction in premalignant cell death and more rapid leukemic transformation (116). Similar to human MDS, the *NHD13* mice (expressing the *NUP98-HOXD13* fusion from the hematopoietic *Vav-1* promoter) display cytopenias, bone marrow dysplasia, and increased HSPC cell death, and the mice die of acute leukemia (predominately myeloid) or bone marrow failure by about a year of life (117). Compared to *NHD13* mice; *NHD13; Tlr2*^−/−^ and *NHD13; MyD88*^−/−^ mice have reduced survival and accelerated leukemogenesis, and mechanistic evaluations showed that loss of TLR signaling was associated with a reduction in activated caspase-1 and decreased cell death of premalignant HSPCs. Furthermore, we noted that while young adult *NHD13* mice displayed increased surface TLR2 expression on their bone marrow HSPCs compared to WT littermates, this expression was markedly reduced (or absent) on leukemic blasts, suggesting that TLR2 is downregulated at some point during the transformation process. This finding is consistent with the human studies showing higher expression of TLR2 in low-risk patients compared to high-risk (69, 70), as well as the correlation between TLR2 expression and cell death (70). Together, these data suggest that while TLR2 signaling may contribute to the death of HSPCs- a prominent feature of low-risk disease- it may also serve a protective role against the accumulation of premalignant cells and ultimate transformation. It is presently unclear what regulates TLR2 expression in the HSPCs of patients with MDS, and how this expression is reduced in higher-risk patients.

### Targeting TLR Signaling in the Treatment of MDS

As discussed above, multiple components of the TLR signaling pathway are upregulated in MDS, which has prompted enthusiasm for targeting this signaling therapeutically. A TLR2 inhibitory antibody (Tomaralimab, Opsona Therapeutics) has now been trialed as second-line therapy for patients with lower-risk MDS (NCT02363491) (118). In addition, a phase I study of a novel small molecule inhibitor of IRAK4 is currently enrolling patients with high-risk MDS and AML (NCT04278768) (119). Of note, enhanced TLR signaling is largely specific to lower-risk MDS, where it promotes cell death, and the effects of TLR signaling and the potential consequences of its inhibition in higher-risk patients is not clear. That said, certain elements of TLR signaling, such as expression of the activating form of IRAK4 (IRAK4-L), are more characteristic of high-risk MDS and progression to AML (76). Thus, not all TLR signaling may be equal in MDS, with certain TLRs or signaling components conferring different effects in different disease contexts. These nuances regarding the role of TLR signaling in both lower- and higher-risk MDS warrant further study, and will help inform which patients might benefit from modulation of TLR signaling, as well as identify the components of this signaling pathway that represent the best therapeutic targets.

Finally, while inhibition of TLR signaling has been the general strategy for MDS, multiple studies suggest that stimulation of this signaling may have therapeutic efficacy in AML, with TLR agonists promoting the death and differentiation of myeloid blasts (120–126). For example, S100A9 was found to induce cell differentiation and growth arrest and prolong survival in a mouse model of AML, and mechanistic studies showed that S100A9 induced this differentiation in AML cells via S100A9 binding to TLR4 and activation of ERK1/2 and JNK signaling pathways (125). Eriksson et al. (124) demonstrated that the TLR1/2 agonist PAM_3_CSK_4_ induces p38-dependent apoptosis and NF-κB-dependent differentiation of both murine and primary human AML cells (124). Similarly, Ignatz-Hoover et al. (123) found that TLR8 activation promotes AML differentiation and growth inhibition in a MyD88 and p38-dependent fashion. Finally, TLR7 stimulation with imiquimod inhibited proliferation, upregulated myeloid differentiation markers, and induced apoptosis in AML cell lines (127).

In addition to directly promoting AML cell death and differentiation, TLR agonists have also been considered for use as anti-tumor agents for their indirect effects on leukemia cells via promotion of the natural immune response to these cells (128–130). For example, incubation of primary AML cells with the TLR7/8 agonist R-848 led to increased expression of MHC molecules, proinflammatory cytokine production, and enhanced stimulation of allogeneic NK, NKT, and T cells (128), and loading of AML cells with the TLR3 agonist polyriboinosinic polyribocytidylic acid [poly(I:C)] led to increased MHC expression, apoptosis, and proinflammatory cytokine production, and enhanced the maturation and activation of dendritic cells after their uptake (125, 128, 129). Thus, the role of TLR signaling in MDS appears to be context-dependent, and the clinical application of agents that modulate TLR signaling should take into account the disease status, likelihood of progression to AML, and the particular components of the TLR signaling machinery that are activated in a given patient. Finally, TLR-targeted therapies alone may not eradicate MDS, but may be a useful component of therapy to optimize normal blood cell production and slow disease progression.

## Summary and Future Directions

**Figure 1:**
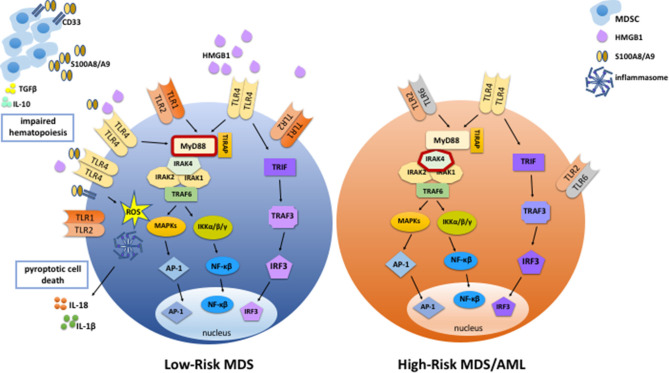
TLR signaling is enhanced in MDS. In low-risk MDS, TLR signaling is enhanced via increased expression of TLRs, increased abundance of DAMPs such as HMGB1 and S100A8/A9, and increased sensitivity to TLR ligands. S100A8/A9 binds to TLR4 and CD33, stimulating inflammasome assembly and pyroptotic cell death by promoting the production of inflammasome components, ROS, and proinflammatory cytokines such as IL-1β and IL-18. In addition, S100A8/A9 binding to CD33 expands MDSCs, which contribute to the suppression of normal hematopoiesis via the production of cytokines such as IL-10 and TGFβ. Activated MDSCs secrete more S100A8 and S100A9, thereby promoting autocrine and paracrine TLR and CD33 signaling and stimulating further expansion of MSDCs and inflammatory cytokine production. In high-risk MDS and AML, endogenous DAMPs and MDSCs are less abundant than in low-risk MDS, and there is less cell death. Also, expression levels of TLR2, TLR4, and MyD88 are lower than in low-risk MDS. On the other hand, expression of an activating form of IRAK4 is associated with high-risk MDS and leukemic transformation.

Enhanced innate immune signaling, and in particular TLR signaling, is a common feature of MDS. The etiology of this TLR signaling upregulation is multifactorial, involving increased expression of TLRs and downstream signaling components, enhanced sensitivity to TLR stimulation, and increased availability of TLR ligands in the bone marrow and blood of patients with MDS. In many cases, the mechanism of TLR upregulation is not clear. Furthermore, TLR expression differs between low- and high-risk patients, suggesting that the role of TLR signaling may change as MDS disease progresses (Figure 1). Further studies are necessary to determine the contribution of specific TLRs to disease pathogenesis, as well as to understand how their expression is regulated in both lower- and higher-risk MDS. Finally, the contribution of TLR signaling in cell type(s) aside from CD34+ HSPCs to the ineffective hematopoiesis and/or leukemic progression is not well-understood, and a more comprehensive study of how TLR signaling is altered throughout the bone marrow in patients with MDS is warranted. Ultimately, these studies will help to inform the development of improved TLR signaling-targeted therapies for the treatment of MDS.

## Author Contributions

LP and LS wrote this review manuscript and generated the figure together. All authors contributed to the article and approved the submitted version.

## Conflict of Interest

The authors declare that the research was conducted in the absence of any commercial or financial relationships that could be construed as a potential conflict of interest.
